# Climatic niche convergence through space and time for a potential archaeophyte (*Acacia caven*) in South America

**DOI:** 10.1038/s41598-023-35658-8

**Published:** 2023-06-08

**Authors:** Nicolás Velasco, Nicolás Andrade, Christian Smit, Ramiro Bustamante

**Affiliations:** 1 Departamento de Ciencias Ecológicas, Facultad de Ciencias, Instituto de Ecología y Biodiversidad, Universidad de Chile, Santiago, Chile; 2 Groningen Institute for Evolutionary Life Sciences, University of Groningen, Groningen, The Netherlands; 3 Facultad de Ciencias Agronómicas, Universidad de Chile, Santiago, Chile; 4 Cape Horn International Centre, Cape Horn County, Chilean Antarctic Province Chile

**Keywords:** Biogeography, Ecological modelling

## Abstract

Based on the niche conservatism hypothesis, i.e. the idea that niches remain unchanged over space and time, climatic niche modelling (CNM) is a useful tool for predicting the spread of introduced taxa. Recent advances have extended such predictions deeper in time for plant species dispersed by humans before the modern era. The latest CNMs successfully evaluate niche differentiation and estimate potential source areas for intriguing taxa such as archaeophytes (i.e., species introduced before 1492 AD). Here, we performed CNMs for *Acacia caven*, a common Fabaceae tree in South America, considered an archaeophyte west of the Andes, in Central Chile. Accounting for the infraspecific delimitation of the species, our results showed that even when climates are different, climatic spaces used by the species overlap largely between the eastern and western ranges. Despite slight variation, results were consistent when considering one, two, or even three-environmental dimensions, and in accordance with the niche conservatism hypothesis. Specific distribution models calibrated for each region (east vs west) and projected to the past, indicate a common area of occupancy available in southern Bolivia—northwest Argentina since the late Pleistocene, which could have acted as a source-area, and this signal becomes stronger through the Holocene. Then, in accordance with a taxon introduced in the past, and comparing regional vs continental distribution models calibrated at the infraspecific or species level, the western populations showed their spread status to be mostly in equilibrium with the environment. Our study thus indicates how niche and species distribution models are useful to improve our knowledge related to taxa introduced before the modern era.

## Introduction

Climatic niche models (CNMs) are commonly used to examine the relationships between niche requirements and the geographic distribution of species^1^. Their usefulness is particularly evident for plants, where temperature and precipitation are the most important predictors of their distribution^2^. The CNMs have become largely formalized in a theoretical framework proposed in several studies^3–5^, where niches are inspired by the Hutchinsonian hypervolume, with the position of one organism (or species) immersed in a multidimensional abstract space defined by ecological variables relevant for its fitness (E-space). This niche constitutes a complement and a duality with the Grinnellian niche^6^, which represents the geographical space (G-space) and includes suitable conditions for living. Albeit complementary, CNMs at, focusing on the abstract ecological space, have been separated in terminology from species distribution models (SDMs), which mainly address the distribution of species in geography^3,6^.

One way of approaching the complete niche of a species is by looking at its full distribution^7^, for example, in cases where taxa reach new areas, limited earlier by dispersion. Yet, several studies have shown that addressing the whole niche of a species is flawed when using complete occurrence datasets only at the species-level^8–11^. Similarly, when studies compare CNMs for specific species, they only produce projections of partial niches (realized or observed)^12^. Prediction improvements can however be made by merging or comparing specific niche spaces below the species level^13^ (i.e., populations, ecotypes, subspecies).

CNMs have been frequently used to study the biogeography of introduced plant species. In fact, the climatic niche can be considered a species trait to examine in the potential distribution of exotics in a new range^14–16^. Despite evidence indicating that species niche shifts take place at some invasions^17–19^, one basic assumption is that if species conserved their climatic niche through time, exotic species can only colonize climatic analogue regions with similar conditions to their native ranges^2,20,21^. This idea of niche conservatism is important from an evolutionary point of view^4,22^, where niche conservatism is tested by tracing niche and past distributions along phylogeny^12,23–25^.

Recently, the idea of niche conservatism has raised-up again as a conceptual framework in invasion ecology because some species have a long history of invasion that can be traced back to dates before 1500 AD (pre-Columbian times), i.e. before trade around the globe started to rise considerably. This distinction differentiates between “*archaeophytes*”: species that were introduced to a new area before 1492 AD (i.e., before the discovery of the American continent or New World); and “*neophytes*”: species that were introduced after 1492 AD^26,27^. Common examples of archaeophytes are plants used by people for food, medicine, or ornamental purposes^27^, and include amongst others, species of different genera like *Livistona*^28^, *Adansonia*^29^, *Acacia*^30^, and *Lilium* species^31^.

Although archaeophytes are regarded as natives in the “new” areas, the term is useful when investigating the historical period or route of colonization by these taxa^32,33^. Indeed, in many cases, the source areas of archaeophyte populations are unknown, so niche conservatism is useful to track down where these populations came from, with the expectation that niches would keep some features from their past ranges. For example, the CNM of the neophyte range of *Lilium lancifolium* was different from its native range, but it was more similar to its archaeophyte’s range, while the latter was also more similar to its native’s range, suggesting a *stepping-stone* process of niche conservatism and colonization^31^.

The distinction between archaeophytes and neophytes is also important when examining the importance of human disturbance as a driver of the spread. For example, neophytes are common in urban areas, while archaeophytes are frequent in rural settlements^34^. Neophytes are also more frequently found in disturbed areas than archaeophytes species^35^, whilst the latter harbor more associations with native pollinators than neophytes due to a longer history of interactions^33^. These examples show the importance of including a temporal dimension when studying introduced species. Similarly, the comparison of introduced and native range niches also benefits from considering the temporal dimension, as the comparison of niches depends largely on the stage of the spread^36^. However, niches can be totally different if dispersal stages are not equal^31^. Gallien et al.^7^ created a comparison framework between the local and the global distribution models (i.e., from occurrences in a specific area, or worldwide, respectively) projected in the introduced areas, which has been widely used on invasion ecology^37,38^. In this framework, four possibilities of spread status could be estimated: adaptations, if the populations in the new range were only predicted by the local model; colonization, if they were predicted only by the global model; sink, if they were poorly predicted by both models; and stabilizing population, if they were predicted accurately by both models (i.e., species or populations are in equilibrium with the environment). Given that older introductions usually are in equilibrium with the environment in the introduced ranges (i.e., the species has already reached all suitable areas)^7,39,40^ one could expect that for archaeophytes, as being long-time present in the “new” ranges, most populations should be in a stabilizing stage of spread.

An interesting example of an archaeophyte plant species is *Acacia caven* in South America, which inhabits a subtropical range at the east of Andes^41^, with humid summers and dry winters, and a Mediterranean range at the west of Andes (Chile), with dry summers and wet winters^42^. Several authors consider the species a probable anthropogenic introduction from the eastern to the western range (Chile)^43–46^. The movement of old tribes and their pack animals are probable vectors of this introduction during the Pleistocene-Holocene period^44^. Support for this idea is found in the broad distributional range across subtropical climate in the South American Chaco region (east of Andes), while the disjunct distribution in the Mediterranean western range seems atypical^41^. Furthermore, no other member of the genus *Acacia* is found in the western range, while there is a large diversity east of the Andes^47,48^. This pattern is also remarkably abrupt considering that plenty of infraspecific variability exists within the species at the east of the Andes^41,49^, while lacking in the western range. Moreover, there are plenty of *Acacia* fossil records in the eastern distribution, but none in Chile^50^.

The origin of *Acacia caven* at the west of the Andes has thus far received little attention. It is therefore unclear where the species came from, why it is present both in the west and east of the Andes where climates are different, and—if considered as archaeophyte—in which status of dispersal-colonization it is. In accordance with the niche conservatism theory, one would expect that even if climates are different, the environmental conditions used by the species will keep mostly the same between the east and west ranges. Consequently, if the species is an archaeophyte, most of its distribution should be in an equilibrium of spread, while if niches are alike, one could profit off of the slight differences to track down the areas of potential origin.

In this study, we tested to what degree the climatic niches (CNMs) of *A. caven* overlap between its eastern and western range, using a set of different metrics. Then, we calibrated optimal species distribution models (SDM) based on the current climatic conditions and transferred them to past Pleistocene-Holocene G-spaces of South America to predict a potential period and source-area for its introduction. Finally, by comparing the predicted archaeophyte’ range distribution model with the continental distribution model (at infraspecific or species level), we tested the status of the spread of *A. caven* in Chile.

## Results

### Niche overlap (CNMs)

**Figure 1:**
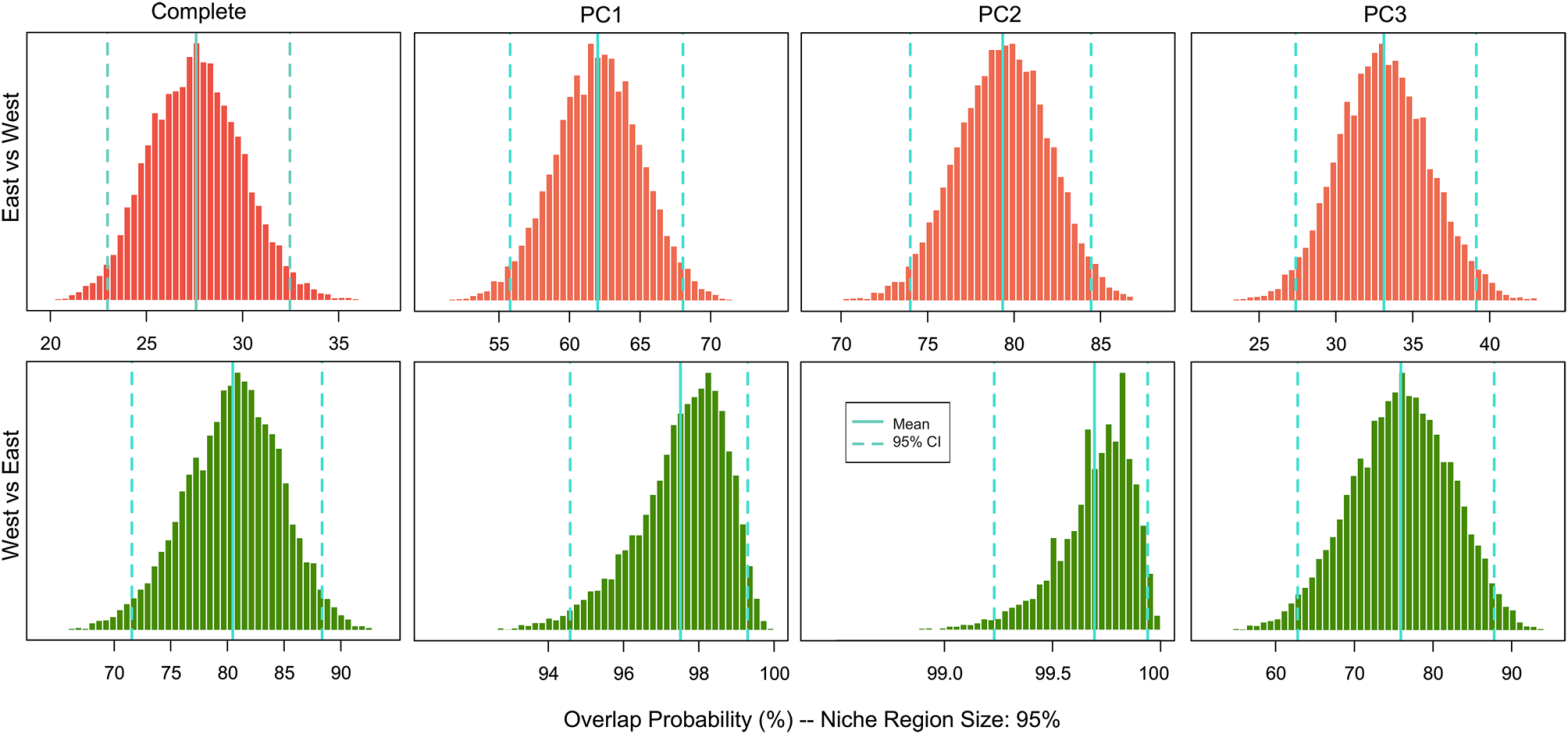
Credible Intervals (CI) for the overlaps in the Three-Dimensional E-space and individual predictors. Interval and mean of the overlap estimation between both ranges, considering a niche region size of 95% of the conditions. Complete: all three PCs altogether. Values estimated after 10.000 Monte Carlo resampling.

The Welch’s t-test indicated that fourteen from the fifteen variables selected had significant differences on the means between both regions (Sup. Table 2). Yet, the climatic grid analysis showed that both niches are similar considering the complete background conditions but are not totally equivalent on the use of them (west–east comparison; similarity convergence p-value = 0.01, equivalency divergence *p* value = 0.01) (Sup. Fig. 1, Table 1). The first two dimensions explained almost 80% of the climatic variability, with many conditions in the background environment not being used. Focusing on the suitable conditions for the particular species ecotype, there is larger variability in the eastern range, while for the western range (Chile), almost all the conditions used are already present in the east (Sup. Fig. 1). Despite the slight difference in the E-spaces used, the western range niche is almost completely covered in the eastern one (Stability = 0.967, Table 1). The results of the E-space in three dimensions showed that the overlap is kept even when adding a new axis of analysis (Sup. Video 1, Fig. 1, Sup. Fig. 2). However, the overlap depends on the regions assessed. For example, in the conservative analysis (NicheA), the eastern region overlaps only 14.04% with the western range, but the other way around, 32.68% of the western niche overlaps with the eastern one (Sup. Video 1, Table 2). When niches are estimated as ellipsoids, the overlaps increase up to 40.3% and 92.5%, respectively, at 99% of CI (Table 2). The projection of different runs of niche intervals showed that independent of the predictors assessed, there was always a large overlap between both ranges (Fig. 1). Between the individual predictors, the third PC had the lowest overlap (Table 2, Sup. Fig. 2).

**Table 1:** Metrics of the climatic grid analysis (two-dimensional E-space) for both ranges of *Acacia caven* var. *caven*.

Comparison		Overlap (D)	Similarity test (*p* value)		Equivalency test (*p* value)		Unfilling	Stability	Expansion
			Less (DIV)	More (CON)	Less (DIV)	More (CON)			
East	West	0.321	1.000	0.020	0.069	0.941	0.413	0.967	0.033
West	East	0.321	0.990	0.010	0.010	0.990	0.033	0.587	0.413

Parameters: overlap (D; 0 = no overlap, 1 = complete overlap), less (DIV) = *p* < 0.05, indicates that niches have diverged (are less similar or equivalent than expected by chance), more (CON) = *p* < 0.05, indicates that niches are conserved (are more similar or equivalent than expected by chance).

**Table 2:** Overlap between both *Acacia caven* var. *caven* ranges in different three-dimensional E-space analyses.

Comparison		NicheA		NicheROVER				
		CPV	% Overlap	CI (%)	3D Niche	PC1	PC2	PC3
East	West	26.44	14.04	95	27.6	62.1	79.3	33.1
				99	40.3	76.0	90.2	47.0
West	East	11.36	32.68	95	80.6	97.5	99.7	75.8
				99	92.5	99.8	100.0	95.8

Comparisons: East–West, how much of the eastern E-space is contained in the western E-space; West–East: vice versa. CPV: Convex polyhedron volume. Shared Volume of CPV: 3.71. CI: Credible interval.

### Species distribution models (SDMs)

**Figure 2:**
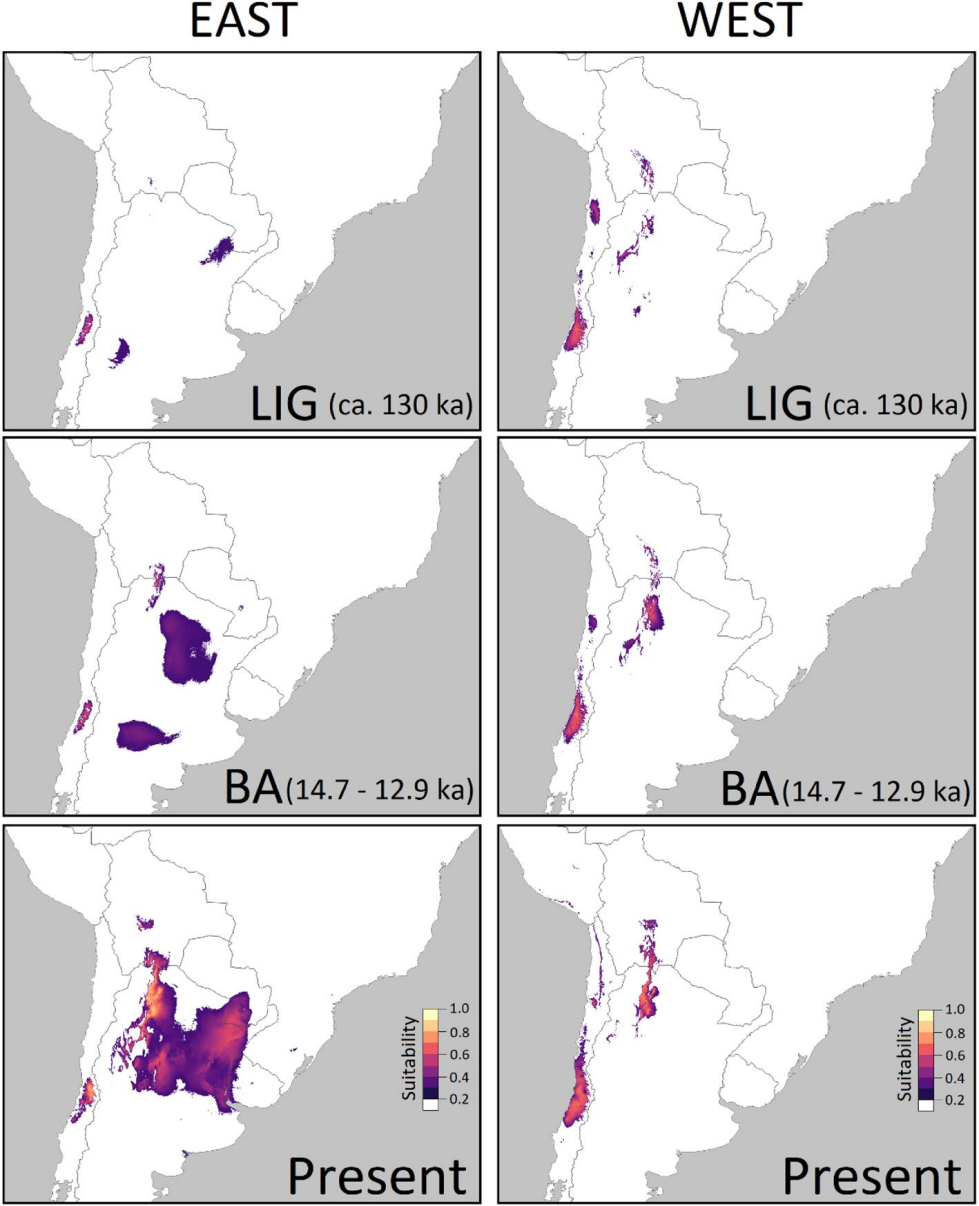
Potential past and current distributions projected from the eastern and western climatic niche models (simplified version). Periods: LIG, Last Interglacial; BA, Bølling-Allerød. For each period, estimated time in ka (1 ka = 1000 years in the past). White = no suitability; Purple areas = lower suitability for the species, yellow areas = higher suitability. Maps generated in QGIS 3.24^51^. All the potential distributions for the other five past periods are available in Supplementary Fig. 3.

The best models selected had slightly different settings of predictors and parameters for predicting each of the four SDMs (Table 3) and both local SDMs were congruent when estimating the current distribution of the species (Fig. 2, Sup. Fig. 3). Archaeophyte and native models transferred to the past scenarios showed that both ranges predicted Central Chile as being always suitable for the particular ecotype presence (Fig. 2, Sup. Fig. 3). Yet, the focus was on eastern areas as potential source for the populations found in Chile. Considering this idea, examining eastern older potential distributions showed that these were constantly far from the western range (Fig. 2. Sup. Fig. 3). Both distributions predict only a small common area at the east of the Andes in the LIG (ca. 130 ka) and some period after the LGM (HS: 17–14.7 ka). Yet, since the Bølling-Allerød period (14.7–12.9 ka), a larger area in south Bolivia—northwest Argentina is predicted by both range’ niches with mid-high suitability, and this pattern becomes more evident through the Holocene (11.7–0.3 ka) (Fig. 2, Sup. Fig. 3).

**Table 3:** Selected models with their parameters and performance.

Region	Sample size	RM	Feature Classes	Set	Mean AUC ratio	Partial ROC	OR < 5%	AICc	Delta AICc	No of parameters	Contribution (%)					
											**PC1**	**PC2**	**PC3**	**PC4**	**PC5**	**PC6**
East	201	0.2	LQP	PC1-6	1.526	0	0.040	4381.699	0.437	25	12.1	19	10.8	16.5	24.5	17.2
West	561	0.1	LQP	PC1-4	1.821	0	0.036	9929.629	0	14	21.4	24.7	46.1	7.8	-	-
Complete (infra)	762	1	LQP	PC1-6	1.468	0	0.047	15.587.1	0	20	4.4	7.1	26.3	3.4	5.7	53.1
Complete (species)	1241	0.1	LQP	PC1-6	1.480	0	0.045	27.503.8	0	25	10.4	15.4	7.4	5.8	18.1	42.8

RM (Regularization Multiplier): controls overfitting of the data; Feature Classes: control complexity of the models, Set: principal components variables used; Mean AUC ratio (mean area under curve ratio): 0 = random performance, 2 = perfect fit; partial ROC (Receiver Operating Characteristic): significance of the model (*p* < 0.01) OR = Omission Rate, check if when models are tested occurrences with errors are less than 5%; AICc = Akaike information criterion corrected for small samples: Delta AICc = difference between model and minimum model (< 2 minimize model complexity).

### Spread stage

**Figure 3:**
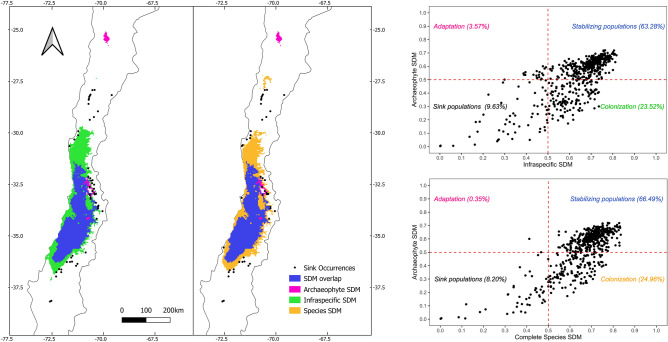
Comparison between archaeophyte (local) SDM and continental SDMs for *A. caven.* Left panel: comparison with an infraspecific distribution SDM and a SDM at the species level. Specific potential distribution predicted by only one model are depicted in different colours. If both models overlap, area depicted in blue. All distributions depicted with a threshold suitability higher than 0.5. Right panel: ubiquity of each western (archaeophyte) occurrence in the dispersal stage, top: compared to the continental-infraspecific model; bottom: versus the continental-species model. Four categories are divided in suitability threshold of 0.5. Sink populations ubiquity also shown in the left maps. Maps generated in QGIS 3.24^51^.

When the two continental SDM (Table 3) were compared to the western range SDM (archaeophyte) (Fig. 3), the results showed that most of the western populations are in a stabilizing status of spread (equilibrium), or in the colonization phase. Adaptive populations were few, while sink populations are present at the borders of the western range.

## Discussion

Here we evaluate the niche overlap between both ranges of the South American tree *Acacia caven*, which is considered an archaeophyte. Accounting for its infraspecific delimitation, we compare its current use on climatic conditions, showing that despite different climates at both sides of the Andes, the species uses almost the same conditions within both ranges. Then, both potential past distributions predict a suitable common area at south-Bolivia northwest-Argentina that may act as source for the spread to west of Andes. Finally, as expected for a potentially long-standing introduction, the spread status showed that almost all western populations are on stable status with the environment.

The results show that the current western niche is similar to, and overlaps largely with, the eastern niche, but that overall conditions are not equivalent (Tables 1, 2, Fig. 1, Sup. Table 2). The overlap estimation depends on the type of analysis performed and which range is considered as a base for the comparison. For example, the Schoener’s D index, which is calculated using the prediction for each of the environmental cells (grid)^52^, showed the most conservative overlap (32.1%). The three-dimensional approaches (NicheA and NicheRover) showed that the overlap with the eastern ranges niche is medium to high, ranging from 32.68% to 92.5%, respectively (Table 2, Fig. 1). Also, this outcome remains unchanged when considering individual variables that summarize the climatic variability (Fig. 1). The findings are not in accordance with a niche shift i.e., use of a large set of different conditions, that could be expected because of the different climates in both ranges^41,42^. On the contrary, the niche dynamics indices indicated that when *A. caven* moved from the eastern to the western range, it filled almost all the niche space (Table 1, Stability = 0.96). These results indicate that the most suitable areas in the western range are already present and used in the eastern range, following the niche conservatism hypothesis^22^. Even though it has already been shown that finding niche conservatism is dependent on the dimensionality used on the analysis^4^, our results are mostly kept even when evaluating the niche at different scales (i.e. two, three, or even individual dimensions). Even though it cannot be ruled out that other niche dynamics may have arisen earlier during the colonization phase at the western range (i.e., niche shifts), the results at least follow the idea that species given sufficient time will track their niche.

When comparing the realized western and eastern SDM (Fig. 2) in current conditions, the western SDM predicts a larger area in the western range than the eastern SDM. Yet, all the new conditions used on the western range, also exist in the environment of the eastern range (Sup. Fig. 1). This could suggest that some biotic interactions restricting the distribution east of the Andes, are not present in the other range. For example, in the eastern range six varieties of *A. caven*^49,53^ and more than 20 species of *Acacia* (today referred as *Vachellia* or *Senegalia*) overlap within the distribution of *A. caven* var. *caven*^47^, which could suggest limited dispersal through some infra or interspecific competition mediated by niche similarities. Yet, follow-up studies would be needed to clearly address how other taxa are involved on the distribution of the species.

Although our analyses lack power to draw causal relationship with the main conditions driving the spread of *A. caven*, the use of principal components boosts generalization as parsimonious models are obtained^54^. Our models are in line with other studies on the natural history of the species that similarly suggested the same common area, where our past niches converged (Fig. 2, Sup. Fig. 3), as a potential source for the populations found in Chile^44^. Interestingly, another legume tree *Prosopis chilensis* has similar patterns across the Andes, with a potential Pleistocene-Holocene introduction in the Atacama Desert (Chile) from populations distributed in the Bolivian-Chaco and Argentine Chaco/Monte regions^11,55^. This indicates that old introduction patterns were not rare events in this region.

The majority of western occurrences, are highly predicted by any model, thus are also in tune with the idea of an old-introduction^7,39,40^. Most of the occurrences were at equilibrium or colonization phase, leaving few areas of the distribution where potential adaptation may be the driving force. Those results are mostly conserved when considering a species level SDM, however the adaptive zones increase when considering an infraspecific level SDM (Fig. 3), which is in line with the notion that models improve when considering the infraspecificity^13^. Together with the results of the equivalency test that partially show some niche divergence (or not strict interchangeability) (Table 2), *Acacia caven* should be considered as a species in equilibrium with the environment, but with an ongoing process of differentiation. For example, in line with the centre-periphery hypothesis^56^ which states that genetic variation and fitness of populations decrease from the centre to the edges of ranges, the adaptive and sink populations for *A. caven* are located at the extremes of the distribution boundaries (Fig. 3). In these few margins, mostly in the dry north or cold foot of the Andes, is where new adaptations might be occurring.

**Figure 4:**
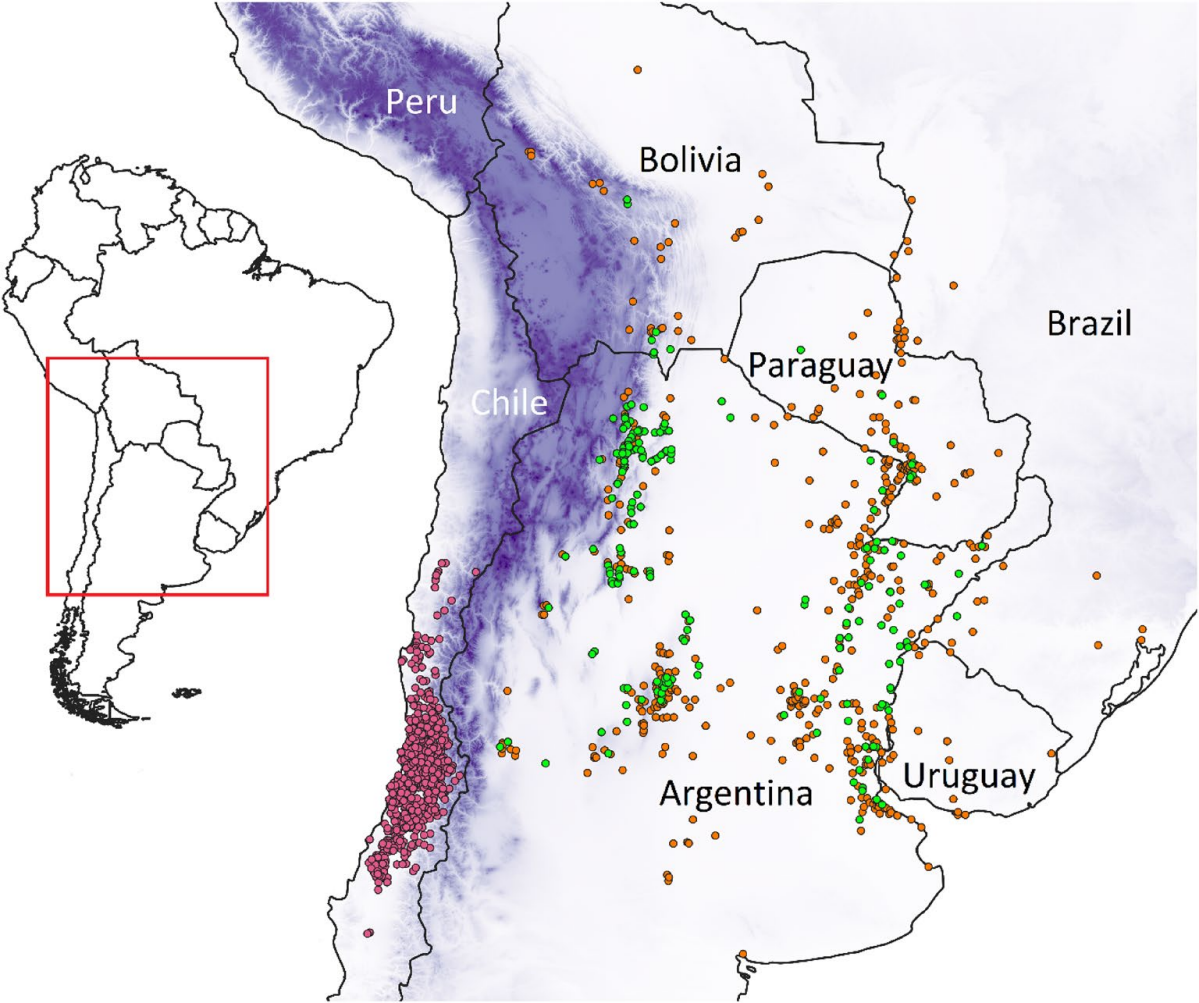
Study areas and occurrence data. Occurrences: pink dots correspond to the western (archaeophyte) range, while green ones are in the eastern (native) range. Orange dots, correspond to additional eastern occurrences at species level or for other infraspecific varieties. Occurrences separate at least ~ 4.6 km between them. Blue represents the elevation of the Andes mountains separating both ranges; the darker the colour, the higher the elevation. White = 0, Dark blue = 6000 m.a.s.l. Maps generated in QGIS 3.24^51^. Elevation data at 15 arcsec from^59^.

A potential weakness in our study is that part of the data could be subject to temporal bias (Sup. Table 3). However, the used data has been selected because it corresponds to adult plants. No regenerating *A. caven* individuals (i.e., younger) were used, which might be using conditions not overlapping the temporal range of WorldClim 2.1^57^. Then, our selection of adult plants’ occurrences must produce more suitable CNMs, as already been suggested by the literature^16^. Additionally, a potential spatial bias could exist in Chile, considering the occurrences clustering (Fig. 4). Still, the data used represent the actual distribution of the species^58^, and the thinning process left only one occurrence matching a specific climatic setting. In accordance, even when eastern occurrences increased to a higher sample size than the used for the western data (i.e., comparison with the complete-species model), the results almost null varied for the dispersal stages (Fig. 3).

When the historical frame of introduced species is accounted for, the impact of alien flora can be reshaped. For example, studies reflect that indigenous people recognize archaeophytes as part of their native flora with positive socio-cultural impacts^29,31^. Nowadays, archaeophytes are even considered important for restoration ecology. For example, despite that the trees *Adansonia digitata* and *Lawsonia inermis* seem to have been introduced in the Middle East before 1500 BP, they are included in ecosystem restoration programs for their importance in agroforestry and for sociocultural purposes (e.g., fuel, dye or antiseptics)^60^. Consequently, considering the niche overlaps, SDMs and dispersal stage results, we should consider *A. caven* as a long-introduced inhabitant in the western range, but not claim that the species is an invasive species with harming effects on its environment. Instead, its dominance in the system and its positive effects on the composition and regeneration of other plant species^44,61,62^, invites us to look at the species as a positive ecological legacy of human-influenced landscapes^63^.

Archaeophyte delimitation is a complex process, and different kinds of independent data (e.g., morphology, ecological niches, and genetics) are needed to unravel the different scenarios and track down potential source areas of their introduction^60,64,65^. For example, using integrative genetics, morphology, and ecological niche modelling, Hardion et al.^65^ suggest that *Arundo donax* is an archaeophyte introduced from Iran to the Mediterranean Basin. While our niche analysis does not provide causal evidence for the introduction of the species, our results are in support of the idea that human-mediated dispersion is the most parsimonious explanation for the presence of *A. caven* in Central Chile. Our finding, that the past species distribution models converge through the late Pleistocene – Holocene period, is congruent with the timespan of the first human settlements in South America^66^. Further studies assessing differentiation of both ranges of *A. caven* using another type of data (i.e., morphology or genetics) would be of value as additional support.

## Conclusions

Despite the discrepancy between climates in the western and eastern range of *Acacia caven* var*. caven*, the ecotype uses conditions with large niche overlap, suggesting niche conservatism with a slight process of divergence. The projection to the past of two different niche models calibrated in both distributional ranges of South America, suggest a potential common area in south Bolivia—Northwest Argentina. The timespan for this area begins in the late Pleistocene and the convergence for western and eastern SDMs becomes more evident in the Holocene. Currently, the spread of the species in the western range seems to be stable, and the species is already occurring in most of the areas that contain the suitable conditions. All in all, the results are in tune with *A. caven* as a potential archaeophyte in Central Chile.

## Methods

### Occurrence data selection and preparation

We obtained *A. caven* occurrence data for its complete distribution area, separating between the western range (Chile)—the potential archaeophyte range, hereafter referred as “WEST”—and the eastern range (Argentina, Bolivia, Brazil, Paraguay and Uruguay), the native range hereafter referred to as “EAST” (Fig. 4). Occurrences from herbaria records were collected physically from different institutions (Sup. Table 1). Additional occurrences were obtained from specific literature reviewing (Sup. Table 1), via the Global Biodiversity Information Facility (GBIF)^67^, and through field surveys in Argentina, Chile and Paraguay during 2019/2020^68^. From the herbaria and field surveys, we selected only occurrences of adult plants in natural areas (i.e., tall trees that main indicates is presence for the last few decades), as previous literature has shown those data as more suitable for CNM^16^. All the data collection was in compliance with relevant national and international guidelines and regulations, and collected herbaria vouchers were deposited in Argentinean and Chilean institutions.

Although the last taxonomic nomenclature has coined *Vachellia caven* for *A. caven*^69^, most local taxonomic studies still use the older name to account for the infraspecific variability^41,47,49,70^. As the names of varieties have not still transferred to the species' new name, but these are important for segregating the datasets in this study, we followed the older nomenclature. Hence, during data collection the infraspecific taxonomy was checked when possible using the fruiting and vegetative traits keys proposed by Aronson^41^ and Pometti et al.^49^. This applies to herbaria records, field sampling and occurrences from GBIF when there was a photo backup (i.e., digital herbaria; iNaturalist). We only kept occurrences that had sufficient information to discriminate them as *Acacia caven* var. “*caven*”. This distinction was important to filter eastern occurrences that contain six varieties^70^, while all western occurrences can only belong to the “*caven*” variety (the only occurring type in this region)^41^. Only for Chile, given the frequency and dominance of the species in the region, we also include data obtained from the landcover dataset of the Chilean National Forestry Corporation^58^. The dataset includes regional landscape shape files classified at the species or vegetation level, with data collected though a vegetation cadastre between 1993 and 1997, and with two categories “*Acacia caven*” and “espinal” (the common/local name for *Acacia caven* landscapes) being suitable for our analyses. Then, these shape files are a proper proxy of the true distribution of the species in Chile. The forest-type images were unified in one shape file, and 400 random points with at least 5 km distance in-between were extracted as additional western occurrences (approx. 2.5 arcmin resolution). This procedure was done with QGis 3.16. As a final preparation step, data were separated as eastern and western distributed, and duplicates per pixel were deleted, using the package “SpThin”^71^ in Rstudio v. 4.0.2^72^. Our thinning process left us with 561 and 201 infraspecific occurrences for the western and eastern ranges, respectively (Sup. Table 3). Prior to the last step in our subsequent methods, we merged both sets to have a complete infraspecific dataset at the continent scale, and finally we also included the discarded eastern occurrences at species or other varieties level (thinned through the same procedure), to obtain a complete continental-species dataset, with 1241 occurrences across the distribution. In this way, four dataset were used for our analyses: local western-archeophyte *A. caven* var. *caven* occurrences, local eastern *A. caven* var. *caven* occurrences, continental-infraspecific (all *A. caven* var. *caven* occurences), continental-species (all *A. caven* occurrences).

### Environmental data

As our first purpose was to elucidate the niche overlap between western and eastern range, under the current conditions, we used the conventional WorldClim bioclimatic variables^57^ at a 2.5 arcmin pixel resolution (~ 4.6 km per side) (Sup. Table 2). From the 19 variables available, bio8, bio9, bio18 and bio19 were discarded as previous research has shown that abrupt patterns of discontinuities exist on these layers^73,74^. To test climatic differences between the two ranges, we extracted the values of the bioclimatic variables using the infraspecific occurrences dataset for both ranges, and means were compared with a two-sided Welch’s t-test (Sup. Table 2). The selected fifteen variables were used as current conditions; then the equivalent bioclimatic variables for past conditions were downloaded from PaleoClim^75^ at the same resolution. For the second purpose i.e., predict period and source-area of introduction, we tested reciprocal past potential distributions, by using six periods: late-Holocene “Meghalayan” (4.2–0.3 ka), mid-Holocene “Northgrippian” (8.326–4.2 ka), early-Holocene “Greenlandian” (11.7–8.326 ka), “Younger Dryas Stadial” (12.9–11.7 ka), “Bølling-Allerød” (14.7–12.9 ka), "Heinrich Stadial" (17.0–14.7 ka), and Last Interglacial (ca. 130 ka). Conditions older than these were excluded as literature claims that the potential arrival of *A. caven* in Central Chile was at the earliest around Pleistocene-Holocene^44^. Also, not all the variables are available for older periods^75^.

### Analyses

**Figure 5:**
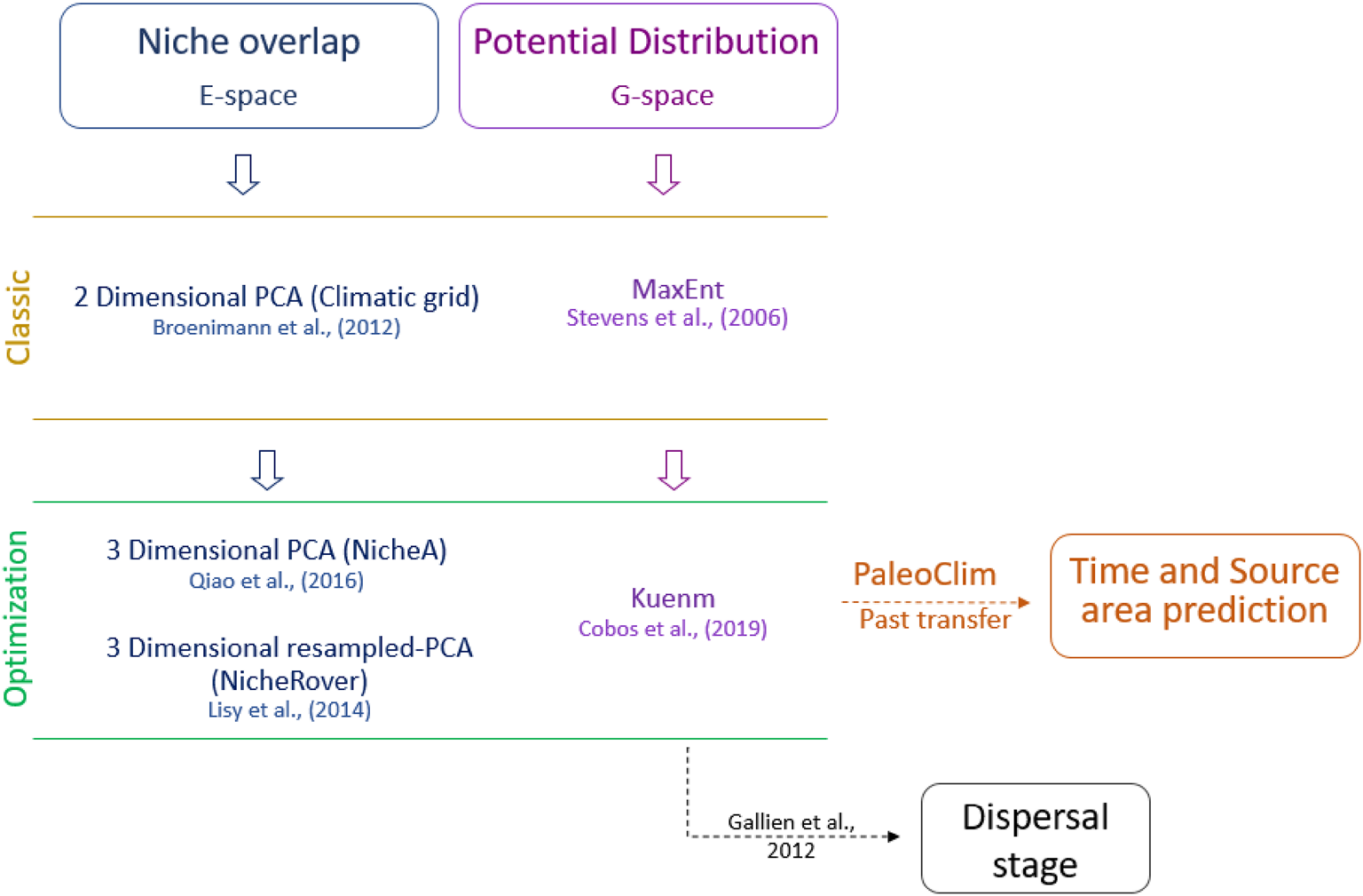
Schematic representation of the analyses. Four main analyses are shown (colors). In the left columns two ways of estimating niche overlap (blue) and potential distribution (purple) are shown: a classic way (yellow row), where estimations are executed only once with the original data and environmental layers, and an improved way (green row) were evaluation of niche overlaps and species distribution models are made through a resampling and an optimization procedure, respectively. *Kuenm* SDM were used as a base for estimating a period and source area (orange) in the past transfer, and for obtaining current suitability values to estimate dispersal stage (black).

We followed the procedure as sketched in Fig. 5. This procedure prevents the bias associated with the classical method, where only one result is estimated from the original data. For example, bias occurs when defining a priori MaxEnt models or estimating niches through a two-dimensional space, that will show overlaps that may not exist using a third dimension. Instead, the improved procedures execute a resampling method for an n-dimensional niche estimation and an optimization procedure in MaxEnt for selecting the best models (between hundreds) to estimate the distributions.

### Niche overlap (CNMs): two-dimensional PCA

Firstly, we estimated the environmental space by using the infraspecific occurrences and the 15 current biovariables selected to construct background E-spaces representing all the countries in the *A. caven* distribution (west: Chile; east: Argentina, Bolivia, Brazil, Paraguay and Uruguay). Following the methods of Broennimann et al.^76^ (Fig. 5), we constructed a PCA for the background conditions of each region. The new climatic conditions were coarsened in 100 × 100 cells using the first two PCs as axes, representing 79.2% of the climatic variability. Each cell represents a unique combination of the environmental envelope. The occurrence data were converted to occurrence densities using a kernel function to smooth them and then projected on east and west background conditions to assess overlap and find which conditions are more frequently used. To estimate niche resemblance, we estimated a series of metrics for comparison, as used in^31^. These included: (1) the Schoener’s D index, which measures niche overlap (0 (no overlap) to 1 (complete overlap)), (2) the equivalency test, which tests if niches are interchangeable (equal) considering only the climate space defined by the exact occurrences, and (3) the similarity test which considers the entire background climatic envelope^77^ and tests if niches are more different or similar than expected by random. The last two metrics were estimated by making 100 replications, with both contrasts (west vs east; east vs west), and two statistical tests (niche divergence or conservation) at *p* value < 0.05. Then we estimated three indices of niche dynamics proposed by Petitpierre et al.^2^: (1) stability, which addresses niche conservatism between A-B ranges; (2) unfilling, e-space used in the A range and not yet occupied in the B range; and (3) expansion, e-space used in the B range and not occupied in A. For each of these three indices the A–B ranges are swapped once (i.e., west vs east, east vs west). All these analyses were performed using the package “ecospat”^78^ in RStudio.

### Niche overlap (CNMs): three-dimensional PCAs

As two-dimensional E-spaces can show an overlap that may be subtle or not exist by including a third axis of variation^4^, we used two approaches to estimate three-dimensional niches. First, we estimated niche as volumes in Niche Analyst (NicheA)^79^ (Fig. 5) using a principal components analysis for all the climatic conditions in South America, and we projected both niches using the first three PCs, which represented 88.4% of the environmental variability (Sup. Table 4). As these variables were used later, before the analyses of the principle components, the current raw conditions (Biovariables-WorldClim) were recalculated in QGis 3.16 to have the same scale units as the corresponding layers from the past conditions (PaleoClim). In NicheA, we decided to express niches as convex polyhedrons that connect extreme occurrences, as these are more conservative than minimum-volume ellipsoids. Then we estimated each range-niche volume and the shared volume to estimate the percentage of overlap. Second, we used a Bayesian approach to estimate confidence intervals of credibility for the overlaps to compare niches as ellipsoids (Fig. 5). We extracted the values for the first three PCs per occurrence, and niches were estimated through 10.000 Monte Carlo resamplings. Finally, overlaps were calculated for the whole niches or the individual PCs predictors, using two niche breadth sizes, alpha = 0.95 or 0.99. The analyses were performed using the package “nicheROVER”^80^ in RStudio. Through this procedure, we avoided bias mediated by differences in occurrence availability (i.e., less at east because of the infraspecific treatment), which can affect the estimation of only one overlap estimation^81^.

### Potential distribution: time and source area prediction (SDMs)

As species distribution models created from CNMs can be produced in different ways^82^, we followed the Cobos et al.^73^ optimization procedure. To reduce environmental collinearity, we used the created NicheA—PCs as predictors, which were also transferred to the six past scenarios. For model creations, we tested 3 sets of current PCs (1–4, 1–5, 1–6) which accounted for approximately ~ 95, 98, and 99% of environmental variability. In each region we calibrated models using a 500 km buffer zone from the occurrences range, to reduce potential overfitting of the CNMs^82,83^. We tested 17 values of regularization multiplier (from 0.1 to 1, at 0.1 intervals; from 1 to 6 at 1 increase interval, 8 and 10), and 31 combinations of features classes (h = hinge, l = linear, p = product, q = quadratic, t = threshold), for model creation, which controls overfitting and complexity, respectively^84^. We created 1581 models for each range through this procedure while selecting the best candidate model was achieved if the model had a significant partial ROC, omission rate < 5% and delta AIC ≤ 2^73,74^. Each model was calibrated using the same 75% of occurrences as training and 25% as a test for the partial ROC and omission rate calculations. Instead, for AIC calculations, all the data were used. Selected models were projected on South America under current conditions to check their consistency and then transferred to the PCs of the six paleo periods selected. This procedure was made with Maxent v. 3.4^85^ with 30 bootstrap replicates for each timespan. As there is a risk of wrong extrapolation when transferring CNMs to past (or future) conditions^73,74^, we decided to not extrapolate the results, and only report the most conservative maps of past potential distributions. All the model creation-selection-&-transfers were performed using the package "*kuenm*"^73^ in Rstudio.

### Spread: status

Following the same procedure as before (*kuenm*) we created additional species distribution models for the current conditions, using the continental-infraspecific dataset and continental-species dataset. Then, using the western occurrences we extracted and compared the predicted suitability values for three species distribution model (SDM) layers (*kuenm* outcomes): local (western -archaeophyte), vs continental-infraspecific or vs continental-species. The occurrences suitability was plotted, separating high and low suitability with a threshold of 0.5 as used in other studies^7,38^. The following four categories used were: sink populations (i.e. occurrences with less than 0.5 in both models); stabilizing populations (i.e. occurrences above 0.5 in both models); adaptation (i.e. high suitability for the local SDM and low suitability in the continental models); and colonization (i.e. low suitability for the local SDM and high suitability in the continental models).

#### Herbaria material revised for occurrences data

Each specimen is cited by the corresponding herbaria abbreviation, the identifier last name and the identification number. In asterisks are denoted the vouchers for the new populations collected in this study.

*Acacia caven* var. *caven*: BAA (Boelcke 411, 571, 3263, 6566, Calderón 1404, Fortunato 2268, Guarnaschelli 71, Hauman 9151, Rua 19337), CONC (Aronson 12097, Finot 157221, Frineso 49328, Lammers 100706, Marticonera 132777, Matthei 106722, Mihoc 165740, Pfister 19724, Quarin 48013, Ricardi 18947, 23963), CORD (Castello 371, Chiapella 2010, Chiarini 1573, Cocucci 3863, 5396, Conci 8492, Demaio 231, Hunziker 4306), CTES (Burkart 152102, Cristobal 256702, 521694, Cuezzo 68182, Krapovickas 108165, 108166, Liesner 106468, Quarin 107768, Schinini 107755, 240191, 107759, Sato 26561, Tressens 107760, 108169, 145613, 146672, 521693, Vanni 55453, 153064, Velasco 0065698*, 0065700*, 0065701*, 0065702*, 0065703*, 0065704*, 0065705*), EIF (Velasco 15268*, 15269*, 15270*, 15271*, 15272*, 15273*, 15274*, 15275*, 15276*, 15277*, 15278*, 15279*, 15280*, 15281*, 15282*, 15283*, 15284*, 15285*, 15286*, 15287*, 15304*, 15305*), LPB (Beck 79, 716, Gallegos 140, Killeen 2691, Lara 480, Michel 3566, Solomon 10181), SI (Deginani 221,159, Múlgura de Romero 221906, Tolaba 221972). *Acacia caven* var. *dehiscens*: CORD (Cerana 1985, Hieronymus 150, Hunziker 57008, 57014, Luti 4434, Martínez 764, Stuckert 1272, Subils 186), CTES (Krapovickas 105690, Luis 231515, Velasco 0065696*, 0065697*, 0065699*), EIF (Velasco 15288*, 15289*, 15290*, 15291*, 15292*), SI (Burkart de Hall 222779, Hurrell 221975). *Acacia caven* var. *macrocarpa*: CTES (Sato 106908, Vanni 256703), LPB (Beck 188, Nee 54917, Solomon 15483, Ritter 1950, Vargas 805), SI (Cialdella 55576, 74763, 74764, 102521, 102522, 102523, 102524, 102525, 102526). *Acacia caven* var. *microcarpa*: BAA (La Porte 2638), CORD (Martínez 299), CTES (Arenas 53434, Aronson 111299, Burkart 521698, Scarpa 390400, Vanni 156781, Velasco 0065706*), FACEN (Vogt 315), FCQ (53927), EIF (Velasco 15298*, 15301*), SI (Arenas 55578, Burkart 206388, 222780, Guaglianone 102513, Ragonese 222783, Rojas 206386, Scarpa 221970, Vega 222037). *Acacia caven* var. *sphaerocarpa*: BAA (Quarín 619), CTES (Aronson 106916, Krapovickas 106918, Quarín 108001, Rumiz 106914, Schinini 107757, Tressens 145472, 145529, 146669, 146675, 146677, Vanni 26055, 111300), EIF (Velasco 15303*), SI (Pometti 54484, 54485, 54486, 54487, 54488, 55579, Ragonese 206399). *Acacia caven* var. *stenocarpa*: BAA (Cosato 195), CTES (Ahumada 534429, Arenas 108170, González 410048, Gutierrez 30726, Maturo 413766, Mereles 253083, Navarro 262783, Salgado 325017, Schinini 107756, 133119, Vanni 186449), FACEN (Benitez 1460, Vogt 3657), FCQ (Garcete 44653, Mereles 13198, 23040, 31623, 37566, Soria 1637, Spichiger 25873, Vera 50969), LPB (Navarro 1900), EIF (Velasco 15299*, 15302*), SI (Cialdella 206390, Rojas 206378).

### Research statements

Permissions for data collection inside National Parks was granted through the Corporación Nacional Forestal (Chile)—Authorization N°011/2019, and Áreas Protegidas Argentina—Delegación Noroeste IF-2020–05378540-APN-DRNOA#APNAC.

## Supplementary Information

Supplementary Information 1.

Supplementary Video 1.

## Data Availability

The datasets generated during and/or analyzed during the current study are available from the corresponding author on reasonable request.
