# Supplementary material for: Climatic niche convergence through space and time for a potential archaeophyte (Acacia caven) in South America
Source: Sci Rep. 2023 Jun 8;13:9340. doi: 10.1038/s41598-023-35658-8 (PMC10250544; doi:10.1038/s41598-023-35658-8)
Supplement: Supplementary file 1 — Supplementary Information 1. [file 41598_2023_35658_MOESM1_ESM.docx]

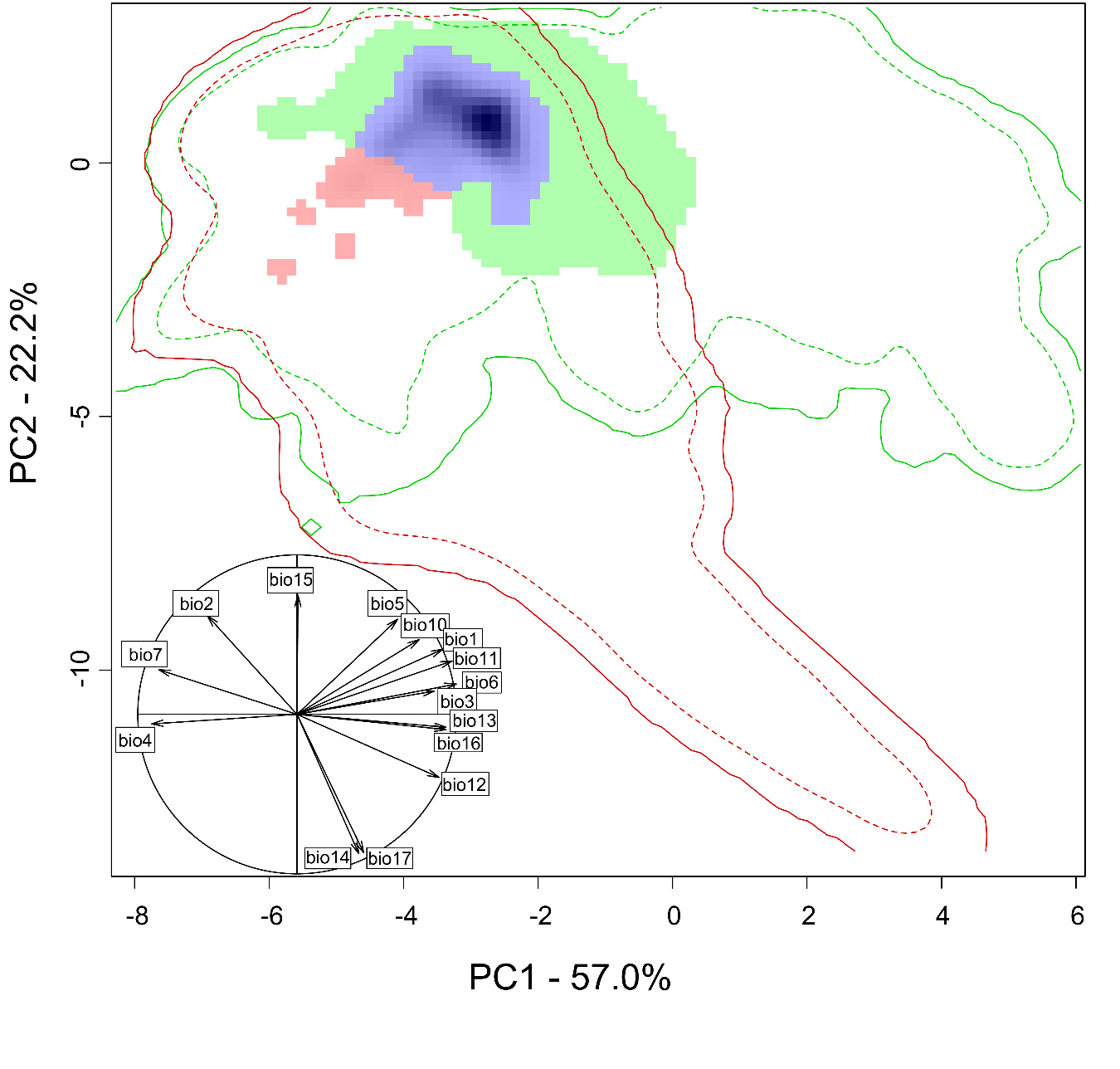
**Supplementary Figure 1 – Two-dimensional E-space.**

Principal component analysis of the climatic space of *Acacia caven* var. *caven* in the native (eastern = green) and archaeophyte range (western = pink). Coloured areas represent conditions that the species uses in each background, while blue areas represent the overlap. Intense colour represents a higher density of occurrences. The solid and dashed lines show 100% and 75% of the background condition per range. The left-lower panel show the contribution of each raw climatic predictor in the PCA.


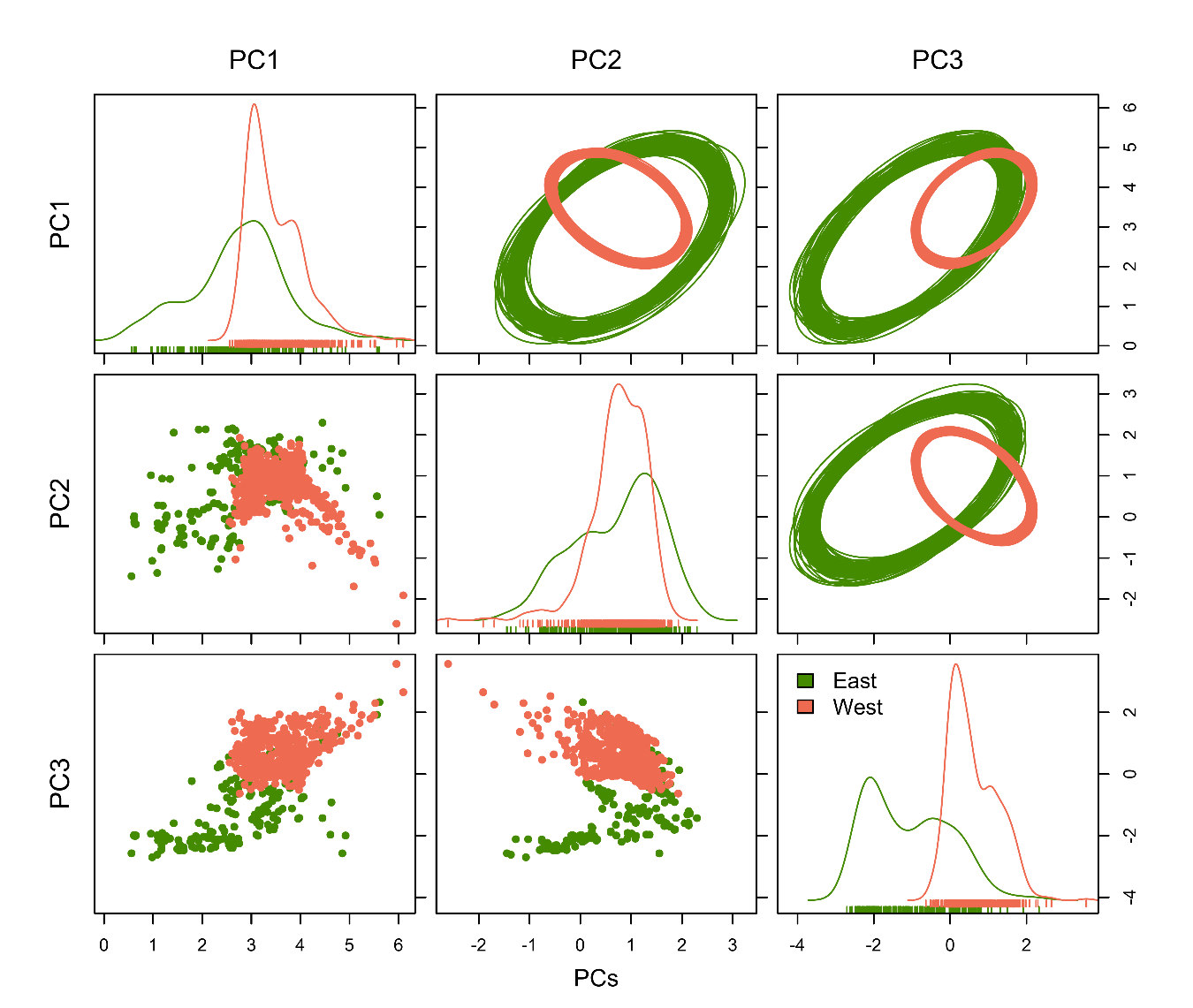


**Supplementary Figure 2 – Summary plots of three-dimensional E-space.**

Dots: raw data for each pairwise PCs combinations. Curves: Density distributions per each PC. Ellipsoids: niche plots, summarizing the Monte Carlo resampling (only 100 for visualization purposes). Green data for eastern range, pink for western.


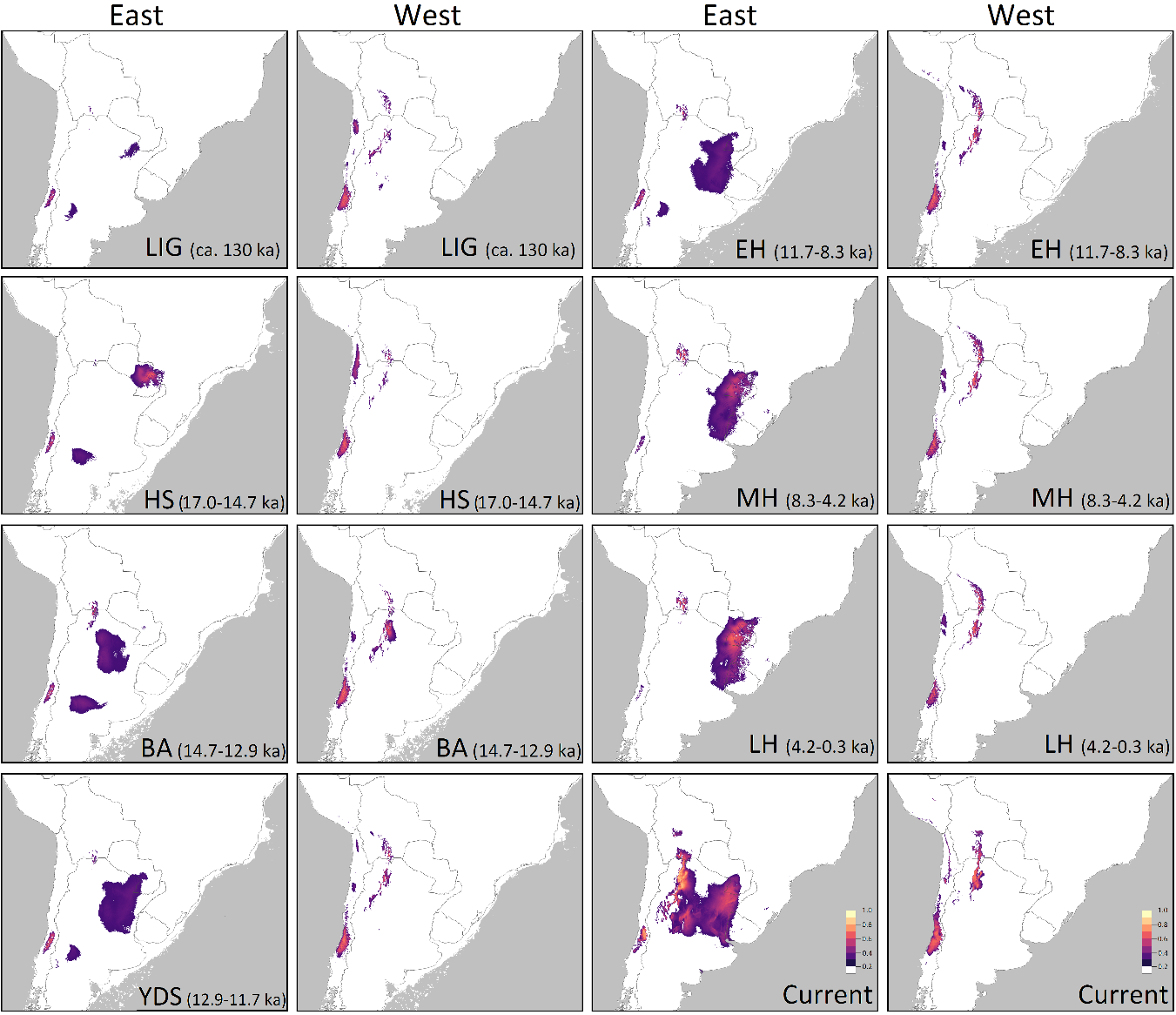


**Supplementary Figure 3 – Potential past and current distributions projected from the eastern and western climatic niche models (large version).** Periods: LIG, Last Interglacial; HS, Heinrich Stadial; BA, Bølling-Allerød; YDS, Younger Dryas Stadial; EH, Early Holocene; MH, Middle Holocene; LH, Late Holocene. For each period, estimated time in ka (1 ka = 1000 years in the past). Purple areas = lower suitability for the species, yellower areas = higher suitability. For simplification, suitability areas lower than 0.2 has been merged with the white background.

**Supplementary Video 1 – Three Dimensional Convex polyhedrons of both ranges into the environmental space performed with NicheA**. Grey dots represent 10.000 combinations of conditions in the first three PCs of the climate of South America. Coloured dots represent conditions used for the species (occurrences), green: in the native (eastern) range; pink: in the archaeophyte (western) range. Polyhedrons connect more distant occurrences.

**Supplementary Table 1 – Institutions (herbaria) and literature sources.**

| ***Herbaria visited*** |
| --- |
| Herbario Gaspar Xuérez (BAA) |
| Universidad de Concepción (CONC) |
| Museo Botánica de Córdoba (CORD) |
| Instituto de Botánica del Nordeste (CTES) |
| Universidad de Chile: Facultad Ciencias Forestales y de Conservación de la Naturaleza (EIF) |
| Universidad de Asunción: Facultad Ciencias Exactas y Naturales (FACEN) |
| Universidad de Asunción: Facultad Ciencias Químicas (FCQ) |
| Herbario Nacional de Bolivia (LPB) |
| Instituto de Botánica Darwinion (SI) |
| ***Literature*** |
| Aronson, J., Ovalle, C., Aguilera, L., & Leon, P. (1994). Phenology of an 'immigrant' savanna tree (*Acacia caven*, Leguminosae) in the Mediterranean climate zone of Chile. Journal of Arid Environments, 27(1), 55-70. |
| Ashworth, L., & Martí, M. L. (2011). Forest fragmentation and seed germination of native species from the Chaco Serrano Forest. Biotropica, 43(4), 496-503. |
| Barberis, I. M., Batista, W. B., Pire, E. F., Lewis, J. P., & León, R. J. (2002). Woody population distribution and environmental heterogeneity in a Chaco forest, Argentina. Journal of Vegetation Science, 13(5), 607-614. |
| Cialdella, A. M., & Novara, L. (1996). Fabaceae-Tribu Acacieae. Aportes Botanicos de Salta-Serie Flora, 4(12), 1-29. |
| Evaldt A., Bauermann, S., & Souza, A. (2013). Descrições morfológicas de palinomorfos holocênicos de um fragmento da Savana Estépica Parque em Barra do Quaraí, Rio Grande do Sul, Brasil. Pesquisas em Geociências, 40(3), 209-232 |
| Ferreras, A. E., Marcora, P. I., Venier, M. P., & Funes, G. (2018). Different strategies for breaking physical seed dormancy in field conditions in two fruit morphs of *Vachellia caven* (Fabaceae). Seed Science Research, 28(1), 8-15. |
| Funes, G., & Venier, P. (2006). Dormancy and germination in three *Acacia* (Fabaceae) species from central Argentina. Seed Science Research, 16(1), 77-82. |
| Giorgis, M. A., Cingolani, A. M., Gurvich, D. E., Reynero, N., & Rufini, S. (2005). Diferencias en la estructura de la vegetación del sotobosque entre una plantación de *Pinus taeda* L.(Pinaceae) y un matorral serrano (Cuesta Blanca, Córdoba). Kurtziana, 31(1-2), 39-49. |
| Hauenstein, E., Muñoz-Pedreros, A., Yánez, J., Sánchez, P., Möller, P., Guiñez, B., & Gil, C. (2009). Flora y vegetación de la Reserva Nacional Lago Peñuelas, Reserva de la Biosfera, Región de Valparaíso, Chile. Bosque (Valdivia), 30(3), 159-179. |
| Karlin, M., Arnulphi, S., Alday, A., Bernasconi, J., & Accietto, R. (2016). Post-fire revegetation in *Acacia* spp. Shrublands in Sieras de Córdoba, Central Argentina. Oecologia Australis, 20(4). |
| López, S. R., Toledo, B. A., & Galetto, L. (2015). Use of wood resources in Central Argentina: A multivariate approach for the study of phytogeography and culture. Ethnobotany Research and Applications, 14, 381-392 |
| Lucero, A., Muñoz, F., Cancino, J., Sotomayor, A., Dube, F., Villarroel, A., & Sáez, K. (2018). Biomass function for *Acacia caven* (Mol.) Mol. distributed in the dry land areas of south central Chile-Función de biomasa para Acacia caven (Mol.) Mol. distribuida en áreas secas del centro sur de Chile. Revista de la Facultad de Ciencias Agrarias, 50(2). |
| Martínez, G., Zagal, V., Ovalle, M., Coûteaux, M. M., Stolpe, N. B., & Valderrama, V. (2010). Descomposición de Hojarascas de *Acacia caven* (Molina) Molina y Lolium multiflorum Lam. en Ecosistemas de Clima Mediterráneo. Chilean journal of agricultural research, 70(3), 454-464. |
| Mereles, M. F., & Rodas, O. (2014). Assessment of rates of deforestation classes in the Paraguayan Chaco (Great South American Chaco) with comments on the vulnerability of forests fragments to climate change. Climatic change, 127(1), 55-71. |
| Planchuelo, A. M., & Ravelo, A. C. (2017). Condiciones termohídricas y comportamiento de especies nativas en el valle de Paravachasca, Córdoba, Argentina. Revista Argentina de Agrometeorología, 8, 43-57. |
| Quiroga, A., & Esnarriaga, D. N. (2014). Diversidad de forrajeras nativas consumidas por el ganado caprino en el área pedemontana del chaco árido, Catamarca. Biología en Agronomía, 4, 121-147. |
| Redin, C. G., Longhi, R. V., Watzlawick, L. F., & Longhi, S. J. (2011). Floristic composition and structure of natural regeneration in Parque Estadual do Espinilho, RS, Brazil. Ciência Rural, 41(7), 1195-1201. |
| Repenning, M., Chiarani, E., Pereira, M. D. S., & Fontana, C. S. (2003). First record of the Chaco Earthcreeper Tarphonomus certhioides (Furnariidae), in Brazil. Revista Brasileira de Ornitologia, 20(4), 453-454. |
| Torres, C., Eynard, M. C., Aizen, M. A., & Galetto, L. (2002). Selective fruit maturation and seedling performance in Acacia caven (Fabaceae). International Journal of Plant Sciences, 163(5), 809-813. |
| Zeballos, S. R., Tecco, P. A., Cabido, M., & Gurvich, D. E. (2014). Composición de especies leñosas en comunidades invadidas en montañas del centro de Argentina: su relación con factores ambientales locales. Revista de Biología Tropical, 62(4), 1673-1681. |

**Supplementary Table 2 – List of WorldClim Bioclimatic variables used and discarded.**

| **Selected initial variables** | | **Mean** | | ***t*** | **df** | ***p*-value** |
| --- | --- | --- | --- | --- | --- | --- |
|  |  | **East** | **West** |  |  |  |
| Bio1 | Annual Mean Temperature | 18.39 | 13.85 | 23.46 | 315.55 | <0.001 |
| Bio2 | Mean Diurnal Range (Mean of monthly (max temp - min temp)) | 12.71 | 12.09 | 6.37 | 374.04 | <0.001 |
| Bio3 | Isothermality (BIO2/BIO7) (×100) | 50.81 | 52.88 | -6.24 | 400.23 | <0.001 |
| Bio4 | Temperature Seasonality (standard deviation ×100) | 439.22 | 404.91 | 6.91 | 333.89 | <0.001 |
| Bio5 | Max Temperature of Warmest Month | 30.32 | 26.86 | 15.65 | 344.42 | <0.001 |
| Bio6 | Min Temperature of Coldest Month | 5.24 | 3.91 | 5.36 | 280.61 | <0.001 |
| Bio7 | Temperature Annual Range (BIO5-BIO6) | 25.07 | 22.94 | 11.86 | 402.61 | <0.001 |
| Bio10 | Mean Temperature of Warmest Quarter | 23.48 | 18.94 | 21.42 | 308.30 | <0.001 |
| Bio11 | Mean Temperature of Coldest Quarter | 12.75 | 8.97 | 18.65 | 311.30 | <0.001 |
| Bio12 | Annual Precipitation | 824.10 | 539.91 | 10.82 | 296.77 | <0.001 |
| Bio13 | Precipitation of Wettest Month | 133.41 | 133.00 | 0.12 | 659.92 | 0.90 |
| Bio14 | Precipitation of Driest Month | 20.55 | 3.48 | 10.58 | 204.59 | <0.001 |
| Bio15 | Precipitation Seasonality (Coefficient of Variation) | 69.93 | 104.48 | -16.02 | 214.49 | <0.001 |
| Bio16 | Precipitation of Wettest Quarter | 361.37 | 340.97 | 2.29 | 650.51 | 0.02 |
| Bio17 | Precipitation of Driest Quarter | 71.81 | 14.60 | 10.44 | 204.85 | <0.001 |
| **Discarded variables** | |  |  |  |  |  |
| Bio8 | Mean Temperature of Wettest Quarter |  |  |  |  |  |
| Bio9 | Mean Temperature of Driest Quarter |  |  |  |  |  |
| Bio18 | Precipitation of Warmest Quarter |  |  |  |  |  |
| Bio19 | Precipitation of Coldest Quarter |  |  |  |  |  |

**Supplementary Table 3 – Temporal range of the western and eastern occurrences used**

| **Temporal Range** | **West** | **East** |
| --- | --- | --- |
| <1970 | 8 | 1 |
| 1970-2000 | 474 | 98 |
| >2000 | 47 | 52 |
| Field work (2019-2020) | 32 | 50 |
| **Total** | **561** | **201** |

**Supplementary Table 4 – Principal components contribution and raw predictors eigenvalues.**

| **PCs** | **Proportion** | **Bio1** | **Bio2** | **Bio3** | **Bio4** | **Bio5** | **Bio6** | **Bio7** | **Bio10** | **Bio11** | **Bio12** | **Bio13** | **Bio14** | **Bio15** | **Bio16** | **Bio17** |
| --- | --- | --- | --- | --- | --- | --- | --- | --- | --- | --- | --- | --- | --- | --- | --- | --- |
| PC1 | 0.568 | -0.294 | -0.244 | -0.314 | -0.296 | -0.302 | -0.165 | 0.039 | -0.302 | -0.177 | 0.206 | -0.271 | 0.285 | -0.200 | -0.325 | 0.288 |
| PC2 | 0.207 | 0.251 | 0.281 | 0.211 | -0.212 | -0.044 | -0.437 | 0.402 | -0.054 | -0.434 | 0.264 | 0.040 | -0.039 | 0.342 | 0.139 | 0.117 |
| PC3 | 0.109 | -0.189 | -0.379 | -0.052 | -0.035 | 0.106 | -0.213 | 0.417 | 0.095 | -0.206 | -0.086 | 0.316 | -0.387 | -0.421 | -0.055 | -0.314 |
| PC4 | 0.055 | 0.087 | 0.083 | 0.095 | -0.325 | -0.385 | -0.115 | -0.273 | -0.391 | -0.127 | -0.556 | -0.012 | -0.077 | -0.039 | 0.201 | -0.330 |
| PC5 | 0.037 | 0.056 | -0.019 | 0.091 | -0.148 | -0.342 | 0.306 | -0.019 | -0.336 | 0.266 | 0.484 | 0.547 | -0.194 | 0.016 | 0.011 | -0.001 |
| PC6 | 0.016 | -0.050 | -0.121 | 0.012 | 0.089 | 0.105 | -0.369 | -0.756 | 0.129 | -0.307 | 0.256 | 0.207 | -0.181 | 0.001 | -0.035 | 0.051 |
